# An Implantable Left Atrial Pressure Sensor Lead Designed for Percutaneous Extraction Using Standard Techniques

**DOI:** 10.1111/pace.12111

**Published:** 2013-02-28

**Authors:** VICTOR PRETORIUS, ULRIKA BIRGERSDOTTER-GREEN, J THOMAS HEYWOOD, WERNER HAFELFINGER, DAN E GUTFINGER, NEAL L EIGLER, CHARLES J LOVE, WILLIAM T ABRAHAM

**Affiliations:** * Department of Cardiothoracic SurgerySan Diego, California; † Department of Cardiac Electrophysiology, University of CaliforniaSan Diego, California; ‡ Ohio State University Division of Cardiovascular MedicineColumbus, Ohio; § Scripps ClinicLa Jolla, California; ¶ Division of Cardiology, Cedars-Sinai Medical Center and the UCLA School of MedicineLos Angeles, California; ** St. Jude Medical, Cardiac Rhythm Management DivisionSylmar, California

**Keywords:** lead extraction, pacemaker infection, left atrial pressure monitoring

## Abstract

**Background:**

An implantable left atrial pressure (LAP) monitoring system for guiding the management of patients with advanced heart failure has the potential to require extraction, particularly in the setting of infection. The LAP sensor lead was designed to be suitable for ease of percutaneous extraction using standard techniques for extracting pacemaker and defibrillator leads. The clinical experience, to date, with percutaneous extraction of the LAP sensor lead is presented.

**Methods:**

A total of 82 patients underwent successful implantation of the LAP sensor lead using transseptal catheterization. Five patients of the 82 patients during a cumulative follow-up period of 267 patient-years (median of 2.9 years/patient) underwent percutaneous extraction using manual traction with a locking stylet and/or an excimer laser sheath to bore through adhesions. The distal fixation anchors of the LAP sensor lead are designed to fold forward during extraction so that the sensor module can easily separate from the interatrial septum.

**Results:**

Percutaneous extraction of the LAP sensor lead was accomplished successfully in all five patients with no embolic events, vascular tears, perforations, or other complications requiring surgical intervention. Manual traction alone was sufficient to detach the LAP sensor lead from the interatrial septum in all cases. Use of the excimer laser sheath was needed in selected cases to bore through scar tissue within the venous insertion site, but not within the heart.

**Conclusions:**

The extraction of the LAP sensor lead was accomplished safely using standard techniques and equipment for percutaneously extracting pacemaker and defibrillator leads.

## Introduction

Implantable hemodynamic monitoring systems for guiding the management of patients with advanced chronic heart failure (HF) have recently been investigated with the aim of reducing hospitalizations for acute decompensated HF.^1^–^4^ An implantable left atrial pressure (LAP) monitor linked to a physician-directed patient self-management treatment paradigm was shown in early clinical studies to be safe with the potential for improved clinical outcomes.^1^ Since the LAP monitoring system, similarly to permanent pacemaker and implantable cardiac defibrillator (ICD) systems, has the potential to become infected, the LAP monitoring system was designed for percutaneous extraction using standard techniques and equipment for extracting pacemaker and defibrillator leads.^5^ In this manuscript we present the clinical experience, to date, with percutaneous extraction of the LAP sensor lead and case histories for two patients who underwent this procedure following an implant duration greater than 2 years.

## Methods

An implantable LAP monitor (HeartPOD™, St. Jude Medical, Sylmar, CA, USA) consisting of a transvenous implantable sensor lead (ISL) connected to a subcutaneous antenna coil was implanted using transseptal catheterization as part of the Hemodynamically Guided Home Self-Therapy in Severe Heart Failure Patients (HOMEOSTASIS) trial.^1^,^2^ Implantation of the ISL is accomplished via transseptal catheterization across the interatrial septum performed from either the femoral venous approach or a superior venous approach. The ISL is loaded into an 11-French delivery sheath and delivered into the left atrium. The delivery sheath is withdrawn to the right side of the interatrial septum and then the ISL is anchored to the interatrial septum using cinching and pivoting maneuvers. Following implantation of the ISL from the femoral venous approach, there is an option to transfer the proximal portion of ISL to a superior venous access site using a custom guidewire that attaches to the connector end of the ISL.^2^

**Figure 1:**
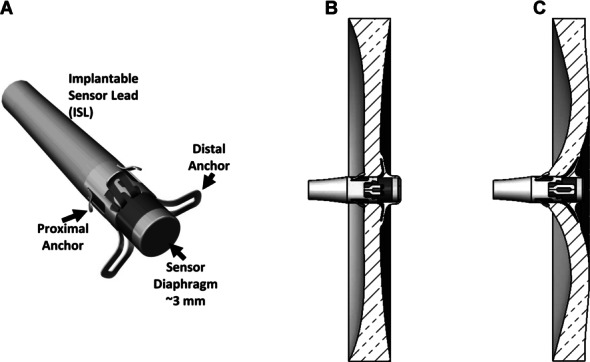
*(A) Illustration of distal tip of the implantable LAP sensor lead showing sensor module with fixation anchors. (B) Illustration of a longitudinal cross section of the sensor module fixated to the interatrial septum with distal fixation anchors covered with tissue and flush with septum. (C) Illustration of a longitudinal cross-section of the sensor module being retracted from the interatrial septum during extraction and causing the distal fixation anchors to fold forward*.

The ISL has a hermitically sealed cylindrically shaped sensor module (3 × 7 mm) located at the distal tip that is affixed to the interatrial septum by proximal and distal nitinol anchors (Fig. 1). The distal nitinol fixation anchors were designed to fold forward upon pulling the ISL from the interatrial septum, while the proximal anchors were designed to have a low profile to minimize the overgrowth of neoendocardial tissue so that the ISL can easily detach from the interatrial septum during extraction. The distal end of the sensor module has a thin titanium-sensing diaphragm that protrudes ∼2 mm into the left atrium. The proximal end of the sensor module has a gradual transition to an 8-French silicone lead body that contains standard inner and outer conductor coils with an internal lumen compatible with a 0.014-inch stylet. The ISL has a ring electrode located 5 cm proximal to the sensor module that is used for acquiring a simultaneous far-field intracardiac electrogram during LAP waveform acquisition. With each recorded LAP waveform, there is also a measurement of core body temperature. The proximal end of the ISL has a standard IS-1 pacemaker lead connector.

HOMEOSTASIS was a prospective, multicenter, observational, open-label registry, comprising the first human use of the LAP monitoring system for the management of chronic HF. The trial was approved by the US Food and Drug Administration (IDE G050018) and the appropriate institutional and ethics committee approval was obtained at each center. All subjects gave written informed consent to participate in the trial. All patients who subsequently underwent percutaneous extraction gave written informed consent to undergo the procedure.

Percutaneous extraction, when indicated, was performed using a stepwise approach using standard techniques and equipment for extracting pacemaker and defibrillator leads.^5^ The ISL was identified and dissection was performed to expose it. Manual traction with the aid of a locking stylet (Liberator™ Universal Locking Stylet, Cook Medical, Leechburg, PA, USA) was initially attempted. If manual traction alone was not successful, then an excimer laser (Model CVX-300, Spectranetics Corp., Colorado Springs, CO, USA), a system using a sheath positioned over the ISL to vaporize adherent fibrotic tissue at the venous entry site, was used under fluoroscopic guidance. Echocardiography imaging guidance was also used in selected cases.

## Results

Between March 2005 and June 2010, 84 consecutive patients underwent implantation of the LAP monitor. The LAP monitor was implanted successfully in 82 patients (98%). The 82 patients were followed over a cumulative period of 267 years with a median follow-up period of 2.9 years/patient. During this follow-up period, five of the 82 patients underwent percutaneous extraction of the ISL. Patient and case characteristics are summarized in Table I. Indications for ISL extraction included suspected infection in four patients and a nonworking implant in one patient. The ISL was extracted successfully in all five cases. A locking stylet was used in four cases, and an excimer laser sheath was used in two cases. There were no major or minor procedure related adverse events and specifically no episodes of stroke or other embolic complications as a consequence of extracting the ISL. There were also no vascular tears or cardiac perforations encountered, or any other complications requiring surgical intervention. Case histories are provided for two patients (No. 1 and 2) in whom ISL extractions were performed following more than 4 and 2 years of implant, respectively. For the remaining three patients, implant duration was relatively short (≤154 days) and percutaneous extraction was similarly accomplished without difficulty.

**Table I:** Patient and Case Characteristics

No.	Age	Gender	EF (%)	Indication for Extraction	Implant Site	Implant Duration (days)	Locking Stylet	Excimer LaserSheath
1	69	F	34	Defibrillator infection	Femoral	1654	Yes	Yes
2	47	M	30	Defibrillator infection	Subclavian	774	Yes	No
3	59	M	35	Defibrillator infection	Subclavian	154	Yes	No
4	48	M	25	Sensor replacement	Femoral	151	Yes	Yes
5	73	F	55	Suspected infected mitral valve prosthesis	Subclavian	77	No	No

EF = ejection fraction.

### Case Histories

#### Case 1

**Figure 2:**
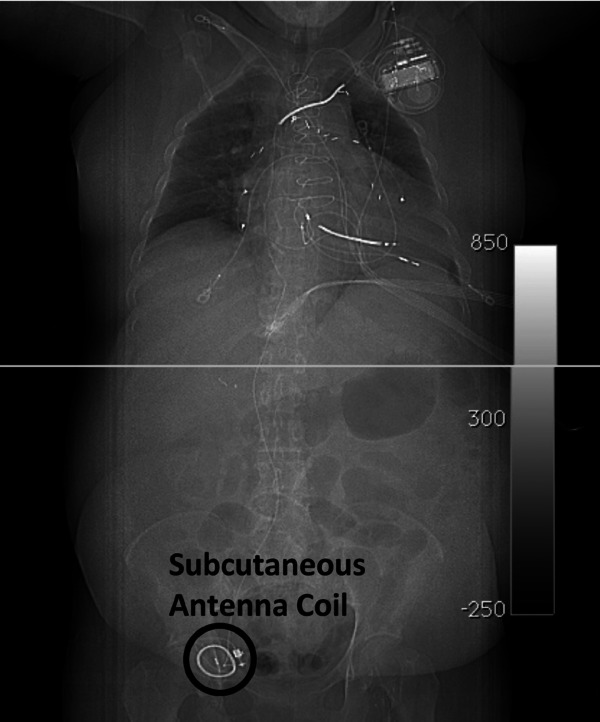
Scout film of chest and abdominal computed tomography scan demonstrating the placement of the ISL and concomitantly implanted pacemaker and defibrillator leads.

The first patient is a 69-year-old female with an ischemic cardiomyopathy who was admitted to the hospital in April 2011 with signs and symptoms of acute sepsis. The patient has a 20-year history of being implanted with pacemaker leads. The patient was first implanted in 1991 with a VVI pacemaker and a right ventricular pacing lead (Passive Plus™, Model 1226T, St. Jude Medical). In 2003 the patient underwent an open coronary artery bypass grafting procedure and during this procedure right atrial (Capsure Epi™, Model 4965, Medtronic Inc., Minneapolis, MN, USA) and right ventricular (Capsure Epi™, Model 4968, Medtronic Inc.) epicardial pacing leads were also implanted. In February 2004 the pacemaker was upgraded to a single-chamber ICD and a right ventricular defibrillation lead (Sprint Quattro™, Model 6947, Medtronic Inc.) was added. In October 2006, the LAP monitor was implanted via the right femoral vein from the inferior approach with the antenna coil implanted within a subcutaneous pocket created above the right inguinal ligament. In April 2010, the single-chamber ICD was upgraded to a cardiac resynchronization therapy defibrillator (CRT-D) and a left ventricular pacing lead was added via the coronary sinus (Fig. 2).

The patient, upon infectious disease workup, was found to have blood cultures growing methicillin-resistant Staphylococcus aureus. The CRT-D pocket was presumed to be infected because there was tenderness and swelling over the device pocket and there was no other available source for the infection. The patient had minimal response to intravenous antibiotic therapy and a decision was made to extract the CRT-D system and the LAP monitor.

**Figure 3:**
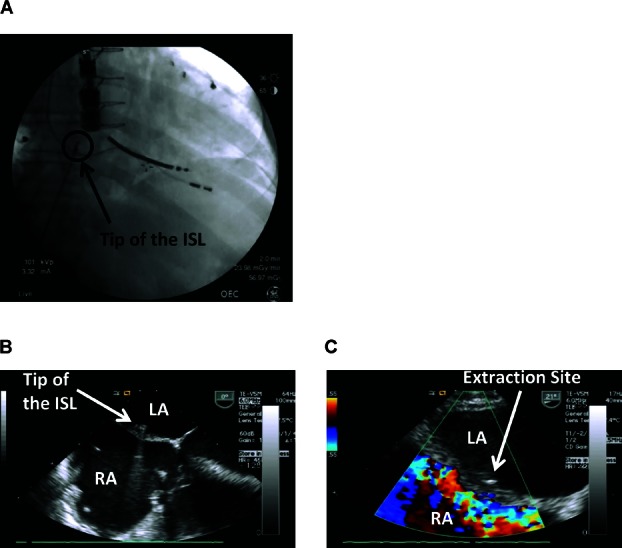
(A) Fluoroscopy image showing manual traction being applied to ISL with a locking stylet via the femoral vein using the inferior approach. (B) TEE image showing reverse tenting while traction is being applied to the ISL. (C) TEE image showing the absence of a residual atrial septal defect immediately following ISL extraction. ISL = implantable sensor lead; LA = left atrium; RA = right atrium; TEE = transesophageal echocardiography.

The procedure was performed in the operating room under general anesthesia using fluoroscopy and transesophageal echocardiography (TEE) for guidance. Echocardiography did not reveal any evidence for intracardiac vegetation or thrombus associated with the LAP monitor. The LAP monitor was removed first, followed by the CRT-D system. Using a single incision over the right femoral vein, the subcutaneous antenna coil was externalized and detached from the ISL. The ISL was then dissected free, and after cutting off the proximal connector end a locking stylet (Liberator™, Cook Medical) was advanced through the open end of the ISL to the proximal end of the sensor module. Using manual traction with the locking stylet, the ISL was extracted easily from the interatrial septum. However, the ring electrode could not be removed from the right femoral venous entry site, and it was necessary to use the excimer laser sheath to allow the ISL to slide out of the vasculature. The extracted ISL had no tissue present over the sensor module or the fixation anchors. However, there was a remnant of a tissue capsule near the ring electrode that necessitated the use of the excimer laser at the femoral venous entry site. The fixation anchors were intact with no evidence of damage. Intraoperative TEE evaluation immediately following ISL extraction demonstrated that no atrial septal defect developed. Additionally, by color flow Doppler, there was no evidence of an interatrial shunt across the site where the ISL was anchored to the septum (Fig. 3).

The CRT-D system was subsequently successfully extracted without difficulty using standard techniques employing a locking stylet and an excimer laser sheath via the left subclavian vein using the superior approach. The epicardial leads were not extracted. The patient tolerated the procedure well without complications.

#### Case 2

The second patient is a 47-year-old male with a nonischemic cardiomyopathy who was admitted to the hospital in October 2010 with signs and symptoms of acute sepsis. The patient had a single-chamber ICD implanted in July 2008, followed by implantation of the LAP monitor in September 2008. Both the ICD and LAP monitor were implanted via the left subclavian vein from a superior approach with two separate subcutaneous device pockets created in the left pectoral region. The patient had recurrent fevers with core body temperature measurements recorded by the LAP monitor up to 39.8°C for 3 days prior to hospitalization, and in-hospital blood cultures growing methicillin-sensitive S. aureus. The ICD pocket had some erythema without fluctuance. A computed tomography scan of the chest showed multiple pulmonary lesions that were suspicious for septic emboli. A TEE study showed a density located on the ISL within the right atrium that was suspicious for possible vegetation. There was no evidence of any thrombus or vegetation within the left atrium. Because of the worsening clinical status and progressive sepsis despite antibiotic therapy, a decision was made to remove the ICD and LAP monitoring systems.

Under intravenous sedation with local anesthesia, the device pockets were opened and all hardware was dissected free of the fibrous tissues and externalized. Under fluoroscopic guidance, the right ventricular defibrillation lead (Durata™, Model 7120, St. Jude Medical) was extracted using direct traction and rotation from the left subclavian superior approach without difficulty. The ISL was then extracted from the interatrial septum with the locking stylet (Liberator™, Cook Medical) using traction alone also from the left subclavian superior approach. The ISL was liberated easily from the septum, and there did not appear to be any other intracardiac or vascular adhesions associated with the ISL. However, upon withdrawing the ISL toward the left subclavian venous entry site, the sensor module could not be removed, and it was necessary to apply counter traction with an 11.5-French polypropylene sheath (Bryd, Cook Medical) to allow the sensor module to slide out of the vasculature. The extracted ISL had a faint amount of a tissue capsule partially over the sensor module and no tissue covering the remainder of the ISL, including the fixation anchors and the ring electrode. There was no damage caused to the fixation anchors. The patient tolerated the procedure well without complications.

## Discussion

This is the first reported case series demonstrating the safety of percutaneously extracting an implantable LAP monitoring system. Percutaneous extraction was successfully performed in five patients using standard techniques for extracting pacemaker and defibrillator leads without encountering any embolic events, vascular tears, cardiac perforations, or other complications requiring surgical intervention. Based on prior experience with extracting pacemaker leads inadvertently implanted in the left ventricle, concerns have been raised regarding the safety of percutaneously extracting the LAP sensor lead.^6^ This is because pacing leads implanted in the left ventricle have the potential to develop left-sided thrombus in the absence of anticoagulation therapy, as well as the potential to develop left-sided vegetation in the setting of infection. The presence of left-sided thrombus or vegetation may complicate the ability to safely perform percutaneous extraction and may require the use of open heart surgery in order to prevent embolic complications during extraction.^7^ In contrast to left ventricular endocardial pacing leads, the implantable LAP sensor lead has minimal protrusion into the left atrium, such that the risk of developing left-sided thrombus or vegetation may be sufficiently minimized to allow for performing percutaneous extraction more safely.

**Figure 4:**
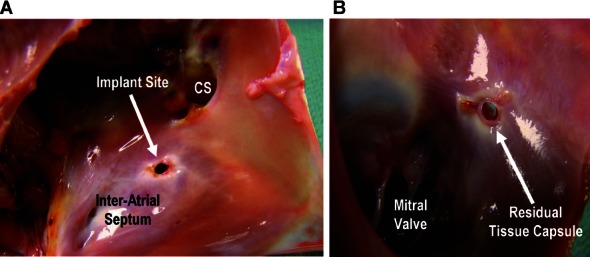
Photographs of the interatrial septum taken from the right side (A) and left side (B) of the septum immediately following percutaneous extraction of the ISL from a canine that was implanted with the ISL for a period of 572 days. Extraction from the septum was performed using the locking stylet alone. The neoendocardial tissue capsule is seen to remain intact over the implant site. CS = coronary sinus; ISL = implantable sensor lead.

Histopathological evaluation in both the preclinical and clinical setting has demonstrated that a thin layer of neoendocardial tissue capsule forms at the distal end of the ISL over the LAP sensor module and fixation anchors, while the remainder of the ISL within the right atrium and the central veins almost always remains free of a tissue capsule and does not tend to form intravascular adhesions. It is hypothesized that the neoendocardial tissue coverage over the distal end of the ISL contributes to the prevention of left-sided vegetation in the setting of an infection. During percutaneous extraction, the ISL is retracted, causing the septum to have reverse tenting and resulting in the distal fixation anchors folding forward and ultimately becoming free from the overlying neoendocardial tissue capsule (Fig. 1). Preclinical experiments using a canine model demonstrate that during percutaneous extraction the LAP sensor module with the fixation anchors detach atraumatically from the septum and the overlying neoendocardial tissue capsule, such that the residual defect created within the septum when present is less than 3 mm (Fig. 4).

**Figure 5:**
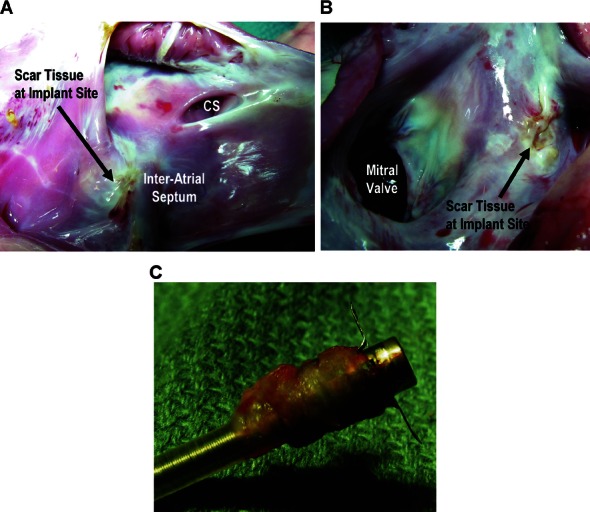
Photographs of the interatrial septum taken from the right side (A) and left side (B) of the septum 28 days following percutaneous extraction of the ISL from a canine that was implanted with the ISL for a period of 723 days. Extraction from the septum was performed using both the locking stylet and the excimer laser sheath. No residual septal defect could be identified at 28 days following extraction on intracardiac echocardiography evaluation and on gross anatomical examination A small amount of scar tissue was found to be adherent to the sensor module following extraction (C). CS = coronary sinus; ISL = implantable sensor lead.

The preclinical experiments also show that whenever a residual atrial septal defect is present following ISL extraction, it has the potential to close over time (Fig. 5), similar to the manner in which iatrogenic atrial septal defects (iASDs) created following transseptal catheterization have the potential to close over time. Singh et al. reviewed the incidence and long-term outcome of iASDs created following transseptal catheterization with a 12-French transseptal sheath in a cohort of 253 patients undergoing implantation of a left atrial appendage closure device.^8^ The reported incidence of a residual septal defect immediately following the procedure was 87% and this was reduced to 7% after 12 months. McGinty et al. conducted a review of the literature on iASDs and found 10 studies, collectively reporting an incidence of residual septal defects of 87% immediately following the procedure and reducing to 15% at 18 months of follow-up.^9^ However, the residual iASDs were well tolerated and not associated with the clinical sequelae of embolism, cyanosis, or right HF. Similar or even lower rates for a residual septal defect created secondary to ISL extraction may be anticipated, since the neoendocardial tissue capsule associated with the sensor module and fixation anchors remains in place following extraction and might provide a tissue cover over the septal extraction site, as illustrated in the first case reported.

Because the primary adhesion formed with the implantation of the ISL is at the distal fixation site to the interatrial septum, a locking stylet that primary engages within the distal end of the ISL is essential. In the cases reported here, manual traction alone with the locking stylet (four cases) or a regular stylet (one case) has been sufficient for extracting the distal end of the ISL from the interatrial septum in all cases. In these cases, no complications were encountered as a consequence of extracting the ISL from the septum. This is unlike a traditional pacing or defibrillator lead that may be attached to the right atrial or right ventricular free wall and/or form intravascular adhesions within the central veins that may potentially result in a perforation or a tear during extraction. The excimer laser sheath was utilized in only two patients to bore through scar tissue that prevented the ISL from sliding out of the femoral venous entry site. Difficulties in sliding the ISL out of the venous entry site may be a consequence of the sensor module having a slightly larger diameter (11 French) in comparison to the 8-French lead body size of the ISL. Application of the excimer laser beyond the venous entry site along the length of the ISL or within the heart was not found to be necessary. This is because the portion of the ISL proximal to the sensor module does not appear to form intravascular adhesions within the central veins. One potential exception might be the ring electrode, located 5 cm proximal to the sensor module, where an adhesion may form to the inferior vena cava when the ISL is implanted from the femoral venous approach. When implanted from the superior venous approach, the ring electrode would usually reside within the right atrium, with no direct tissue contact, and does not appear to adhere.

In this case series it was possible to easily detach the sensor module from the interatrial septum without needing to use the excimer laser to bore through scar tissue formed against the septum. This might have been a consequence of having a small amount of scar tissue develop between the distal and proximal anchors over the relatively short implant duration. In one preclinical experiment (Fig. 5) with a sensor implanted for nearly 2 years it was not possible to use the locking stylet alone to detach the sensor module from the septum, and it was additionally necessary to apply the excimer laser against the septum. The usage of the excimer laser made it possible to detach the sensor module atraumatically from the septum, and the residual septal defect subsequently closed within 28 days.

### Limitations

The number of patients in this case series is small and there were only two patients implanted for more than 1 year. The success rate for percutaneous extraction may be reduced when larger numbers of patients with longer implant durations are studied. All sensor modules were easily extracted from the septum, likely because the tissue capsule formed over the sensor module and fixation anchors was thin, and the distal fixation anchors fold forward, promoting atraumatic detachment from the septum. It is possible that patients with longer implant durations have more complex scar tissue adherent to the sensor, making extraction more difficult and requiring the usage of the excimer laser against the septum. Usage of the excimer laser against the septum may perhaps result in a larger residual septal defect and may increase the risk for embolic complications.

## Conclusion

Extraction of the LAP sensor lead was accomplished safely using standard techniques for percutaneously extracting pacemaker and defibrillator leads. The absence of intravascular adhesions along the length of the ISL, in combination with the ability of the distal fixation anchors to fold forward and easily detach from the interatrial septum during extraction, made it possible to safely extract the LAP sensor lead.
